# Human-specific gene *ARHGAP11B*—potentially an additional tool in the treatment of neurodegenerative diseases?

**DOI:** 10.3389/fmmed.2024.1465647

**Published:** 2024-11-27

**Authors:** Wieland B. Huttner

**Affiliations:** Max Planck Institute of Molecular Cell Biology and Genetics, Dresden, Germany

**Keywords:** *ARHGAP11B*, basal progenitors, human-specific gene, metabolism, neural stem cells

## Introduction

One strategy in the treatment of neurodegenerative diseases has been to replenish lost cells, notably neurons. Approaches taken to this end have included the following: first, to either activate neural stem cells that endogenously exist in certain neurogenic niches of the adult human brain such that new neurons are being generated where needed [for recent reviews, see Temple (2023); Vassal et al. (2024); Velikic et al. (2024)]; second, to graft exogenous neural stem cells and/or exogenously generated neurons into the affected brain region, often by making use of patient-derived induced pluripotent stem cells (iPSCs) to obtain the former cells [for recent reviews, see Lee et al. (2024); Temple (2023); Vadodaria et al. (2020)].

In this brief Opinion Article, I would like to draw attention to the human-specific gene *ARHGAP11B*, which exhibits properties that could potentially be beneficial in the treatment of neurodegenerative diseases.

## Features of *ARHGAP11B*

*ARHGAP11B* is typically referred to as a human-specific gene. This statement is correct in terms of extant species, as *ARHGAP11B* does not occur in any other primate or mammal. However, from an evolutionary point of view, *ARHGAP11B* is actually a hominin-specific gene, as it has been shown to have occurred in Neanderthals and Denisovans, and in light of its origin, ≈5 mya, it likely occurred in other members of the *Homo* lineage [for a recent review, see Huttner et al. (2024)].

Besides the function of the ARHGAP11B protein, that is, to stimulate glutaminolysis in mitochondria (Namba et al., 2020; see *Discussion*), a key feature of the *ARHGAP11B* gene as a potential additional tool in the treatment of neurodegenerative diseases pertains to the cell types in which this gene is expressed. Thus, in the fetal human neocortex, the cells exhibiting the highest level of *ARHGAP11B* expression are the neural stem and progenitor cells. Specifically, during neurogenesis, *ARHGAP11B* is expressed in both the apical progenitors residing in the ventricular zone and the basal progenitors residing in the subventricular zone, notably apical radial glia and basal (or outer) radial glia, respectively (Florio et al., 2015). Such expression can be seen as a strategic advantage if one intends to use cortical stem and progenitor cells for therapeutic approaches in neurodegenerative diseases that aim to achieve cell replacement.

Indeed, and of potential clinical relevance, the expression of *ARHGAP11B* in various animal model systems *in vivo* has been shown to amplify basal progenitors, the progenitor cells that generate cortical neurons (Florio et al., 2015; Kalebic et al., 2018; Heide et al., 2020; Xing et al., 2021). Moreover, the effects of *ARHGAP11B* on basal progenitors result in an increase in cortical neuron production *in vivo* (Florio et al., 2015; Kalebic et al., 2018; Heide et al., 2020; Xing et al., 2021). Of note, *ARHGAP11B* expression *in vivo* increases the so-called upper-layer neurons, the class of cortical neurons implicated in higher cognitive abilities (Kalebic et al., 2018; Heide et al., 2020; Xing et al., 2021). The amplification of basal progenitors *in vivo* by *ARHGAP11B* is based on the ability of this gene to induce basal progenitor self-renewal (Florio et al., 2015; Kalebic et al., 2018; Heide et al., 2020). Hence, *ARHGAP11B* fulfills a key criterion for its potential therapeutic application in neuron replenishment strategies for the treatment of neurodegenerative diseases—the ability to induce *in vivo* the self-renewal of those progenitor cells that generate cortical neurons.

## Potential approaches to using *ARHGAP11B* as an additional tool in the treatment of neurodegenerative diseases

To explore the potential use of *ARHGAP11B* as an additional tool in the treatment of neurodegenerative diseases approaches to be considered include the following. First, one could aim at targeting the endogenous neural stem cells in the adult human brain with an appropriate *ARHGAP11B* expression vector. Neural stem cells and/or neurogenesis in the adult human brain have so far been detected in the hippocampus [for a review, see Kempermann et al. (2015)], the amygdala (Roeder et al., 2022), and the subventricular zone of the lateral ventricles [for a recent summary, see Baig et al. (2024)]. An appropriate *ARHGAP11B* expression vector should feature an inducible on–off expression system to first amplify the respective neural stem cells by switching on *ARHGAP11B* expression and, thereafter, upon switching off *ARHGAP11B* expression, to allow them to generate neurons.

A second line of approach could make use of patient-derived iPSCs that are first converted to neural stem cells, into which an appropriate *ARHGAP11B* expression system is then introduced. Such neural stem cells with the capacity to allow an inducible expression of *ARHGAP11B* could then be administered into the brain region of interest, followed by local neural stem cell amplification and then local neurogenesis, as mentioned above.

## Discussion

Should the transient (i.e., inducible) expression of *ARHGAP11B* indeed lead to local neural stem cell amplification and consequently to local neuronal replenishment, a key future task of this approach will be to determine whether the newly generated neurons are able to functionally compensate for the lost neurons. If so, it may be forward-looking to consider the mechanism underlying the ability of *ARHGAP11B* to amplify neural stem cells. The ARHGAP11B protein has been shown to be imported into the matrix of mitochondria in the cells expressing this gene, where ARHGAP11B stimulates the metabolic pathway called glutaminolysis (Namba et al., 2020). In light of the emerging concept that changes in metabolism exert a crucial impact on the behavior of neural stem cells (Namba et al., 2021), targeting specific metabolic pathways may aid future therapeutic endeavors in the treatment of neurodegenerative diseases.
